# Flavones hydroxylated at 5, 7, 3′ and 4′ ameliorate skin fibrosis via inhibiting activin receptor-like kinase 5 kinase activity

**DOI:** 10.1038/s41419-019-1333-7

**Published:** 2019-02-11

**Authors:** Yifan Zhang, Jing Wang, Sizheng Zhou, Zhibo Xie, Chuandong Wang, Ya Gao, Jia Zhou, Xiaoling Zhang, Qingfeng Li

**Affiliations:** 1 Department of Plastic & Reconstructive Surgery, Shanghai Ninth People’s Hospital, School of Medicine, Shanghai Jiao Tong University, Shanghai, China; 2 Department of Otorhinolaryngology Head and Neck Surgery, Shanghai Children’s Hospital, Shanghai Jiao Tong University, Shanghai, China; 3 Department of Pancreatic Surgery, Huashan Hospital, Shanghai Medical College, Fudan University, Shanghai, China; 4 Stem Cell and Regenerative Medicine Lab Department of Orthopedic Surgery, Xinhua Hospital, Shanghai Jiao Tong University School of Medicine, Shanghai, China

## Abstract

Skin fibrosis is mainly characterized by excessive collagen deposition. Studies have recently identified a number of flavonoids with variable structures that have the potency of inhibiting collagen synthesis and thus attenuating organ fibrosis. In this study, we found that flavones with 5, 7, 3′, 4′ hydroxy substitution reduced collagen expression most efficiently. Among those flavones, luteolin, quercetin, and myricetin were selected for follow-up. In vivo, the three compounds ameliorated skin fibrosis and reduced collagen deposition. Further analysis showed the compounds had significant inhibition on the proliferation, activation and contractile ability of dermal fibroblasts in vitro and in vivo. More importantly, we revealed that luteolin, quercetin, and myricetin selectively downregulated the phosphorylation of Smad2/3 in TGF-β/Smads signaling via binding to activin receptor-like kinase 5 (ALK5) and impairing its catalytic activity. We also found flavones with 5, 7, 3′, 4′ hydroxy substitution showed stronger affinity with ALK5 compared with other flavonoids. Herein, we identified at least in part the underlying molecular basis as well as the critical structures that contribute to the antifibrotic bioactivity of flavones, which might benefit drug design and modification.

## Introduction

Skin fibrosis is the major manifestation of a series of fibroproliferative disorders such as scleroderma, hypertrophic scar (HS) and keloid^1,2^. These fibrotic conditions, in most cases, cause cosmetic, functional or psychological impairment and thus have become a significant social and economic burden^3,4^. For instance, HS is raised and hyperemic, with cicatricial contracture and often leads to debilitated self-esteem and depression^5,6^. Despite numerous studies on the topic, few satisfactory interventions have been put into clinical practice^7^. Therefore, it is of great necessity to find out novel therapeutic targets and develop effective management strategies.

Skin fibrosis is mainly characterized by dysregulated activation of dermal fibroblasts that eventually leads to excessive deposition of extracellular matrix (ECM)^8^. Specifically, substantial collagen expression in the dermis is the most essential and contributes to distorted skin architecture and dysfunction of the skin^9,10^. Although the underlying mechanisms of the disorder is still elusive, researches have revealed that TGF-β/Smads signaling cascade plays a vital role in the initiation and development of such pathophysiological process^11^. Aberrant activation of TGF-β/Smads signaling is associated with exuberant behavior of dermal fibroblasts such as abnormal proliferation and transdifferentiation into myofibroblasts^12–14^. Moreover, Smads downstream of TGF-β are one of the most effective mediators in upregulating the expression of collagen in fibroblasts^15^. Thus, TGF-β/Smads signaling has been a potential therapeutic target for fibrotic disorders of skin.

Flavonoids (from the Latin word flavus meaning yellow, their color in nature) are a class of plant and fungus secondary metabolites. They have been shown to possess a variety of biological characteristics, including antioxidative^16^ and anti-inflammatory effects^17^, tumor growth inhibition^18^, and protection of ischemia/reperfusion injury^19^. Additionally, flavonoids have been proven to alleviate fibroproliferative disorders including cirrhosis, pulmonary fibrosis, and cardiac fibroblasts^20–22^. Also, our group previously discovered a number of flavonoids such as baicalein and galangin that effectively ameliorate HS formation via TGF-β/Smads-mediated inhibition on the proliferation, activation, contractile ability, and collagen production of dermal fibroblasts^23,24^. However, we also noticed a number of flavonoids that had no significant impact on fibroplasia. Recently, it has been demonstrated that the bioactivity of flavonoids is predominantly dependent on the backbones and the functional groups of the compounds, but the decisive structural features for the antifibrotic activities of flavonoids are yet to be uncovered.

In this study, the effect of 109 flavones on the expressions of type I and III collagen was at first detected for the sake of screening for potential agents that might ameliorate skin fibrosis. Furthermore, the therapeutic efficacy of the selected candidate compounds was analyzed in two animal models for skin fibrosis as well as in cultured human dermal fibroblasts (HDFs). Lastly, the molecular basis of flavone-induced inhibitory effect on skin fibrosis was studied and the fundamental structure indispensable to antifibrotic property was verified.

## Results

### Flavones with 5, 7, 3′ and 4′ hydroxy substitution demonstrate significantly stronger inhibition on collagen synthesis than other flavonoids

Chemically, flavonoids have the basic structure of a 15-carbon skeleton that consists of two phenyls (A and B) rings and one heterocyclic (C) ring. This carbon structure can be abbreviated as C6-C3-C6 (Supplementary Figure S1A). Based on their backbones, flavonoids can be divided into several classes: chalkone, isoflavone, flavone, etc. (Supplementary Figure S1B).

**Fig. 1:**
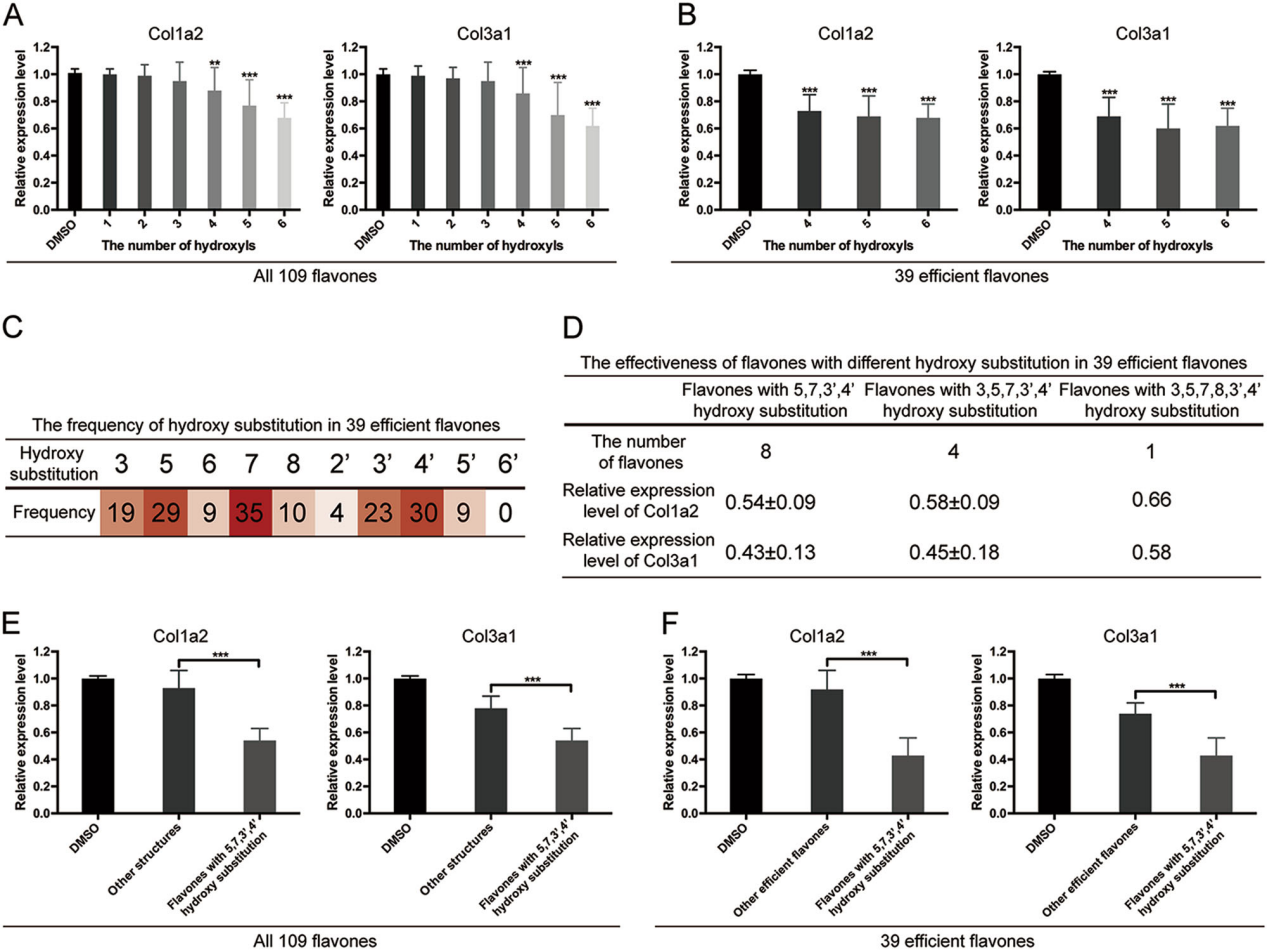
Correlation between hydroxy substitution in flavones and their effectiveness on Col1a2 and Col3a1 inhibition. Flavones with no less than four hydroxyls induce significant inhibition on Col1a2 and Col3a1 expression compared with vehicle in **a** 109 candidate flavones and **b** 39 positive compounds. **c** The frequency of hydroxy substitutions in 39 positive compounds. **d** The effectiveness of flavones with different hydroxy substitutions in 39 positive compounds. Flavones containing 5, 7, 3′, 4′ hydroxy substitution exhibits significantly stronger inhibition on collagen expression compared with flavones with other structures in **e** 109 candidate flavones and **f** 39 positive compounds. Data are the mean ± SD (three independent experiments); **P *<* 0.01, ***P *<* 0.001

One of the most evident manifestations of skin fibrosis is excessive collagen deposition. Thus, to screen for antifibrotic flavonoids, type I and III collagen expressions of HDFs treated with a variety of flavones (10 μM) were quantified by qPCR. We found that higher degree of hydroxylation tended to induce stronger inhibition on collagen synthesis and collagen I and III expression were significantly downregulated by flavones containing no less than four hydroxyls compared with vehicle control (Supplementary Figure S2 and Fig. 1a). Both alkylation and glucuronidation of the hydroxyl groups decreased the capacity to suppress Col1a2 and Col3a1 expressions (Supplementary Figure S1C). These results indicated a potential association between the hydroxyl groups of flavones and their antifibrotic activity. Besides, we also found flavones with different backbones had differing antifibrotic potential. Flavones, which are based on the backbone of 2-phenylchromen-4-one, exhibited the most significant inhibition on collagen expression compared with other classes such as chalcone and isoflavone (Supplementary Figure S1D). Based on the findings above, we focused on 39 flavones with 4, 5 or 6 hydroxyls that significantly reduced collagen expression (positive compounds, Supplementary Figure S2). Firstly, our results demonstrated that tetrahydroxyflavones, pentahydroxyflavones, and hexahydroxyflavones instead of hydroxyflavones and bihydroxyflavones had significantly stronger inhibition on Col1a2 and Col3a1 expressions than vehicle in the positive compounds (Fig. 1b). However, there was no noticeable difference among HDFs treated with tetrahydroxyflavones, pentahydroxyflavones, and hexahydroxyflavones as to the fold changes of Col1a2 and Col3a1 expression (Fig. 1b), suggesting that more than four hydroxyls in flavones might be redundant. The findings above led us to the assumption that the antifibrotic activity of flavones could in part depend on the position of the hydroxyl groups. Among 39 positive compounds, we found that the most frequently hydroxylated position was 7 (35/39), followed by 4′ (30/39), 5 (29/39), 3′ (23/39), 3 (19/39) and 8 (10/39) (Fig. 1c). Further comparison among 5, 7, 3′, 4′-hydroxylated flavones (eight compounds), 3, 5, 7, 3′, 4′-hydroxylated flavones (four compounds) and 3, 5, 7, 8, 3′, 4′-hydroxylated flavones (one compound) showed that the existence of hydroxyl in position 3 (Col1a2 0.54 vs. 0.58, *P* = 0.431, Col3a1 0.43 vs. 0.45, *P* = 0.792) or 8 (Col1a2 0.54 vs. 0.66, Col3a1 0.43 vs. 0.58) did not enhance the inhibitive effect of the compounds (Fig. 1d). This indicated that the most effective and the key hydroxylation pattern was 5, 7, 3′ and 4′ hydroxylation. And indeed, the flavones with 5, 7, 3′ and 4′ hydroxylation was significantly more efficient on downregulating the expression of both Col1a2 and Col3a1 compared with flavones with other structures (Fig. 1e, f). Therefore, flavones containing 5, 7, 3′, 4′ hydroxy substitution were selected for further analysis.

### Luteolin, quercetin or myricetin dose-dependently reduces the expression of type I and III collagen in vitro

To further verify the effect of the positive flavones based on the screening above on collagen expression, luteolin, quercetin, and myricetin that exhibited the strongest inhibitive efficacy were selected for further analysis (Supplementary Figure S3A). Toluidine blue staining of cultured HDFs showed that ECM production was significantly reduced after the cells were treated with luteolin, quercetin or myricetin at 10 μM for 5 days (Supplementary Figure S4A). Furthermore, based on qPCR and western blotting, luteolin, quercetin or myricetin demonstrated specific and dose-dependent downregulation of type I and III collagen expressions in HDFs (Supplementary Figure S3B-D), while the compounds did not induce remarkable changes in other collagen (type II or type X) expressions (Supplementary Figure S4B).

### Luteolin, quercetin or myricetin attenuates skin fibrosis in vivo

**Fig. 2:**
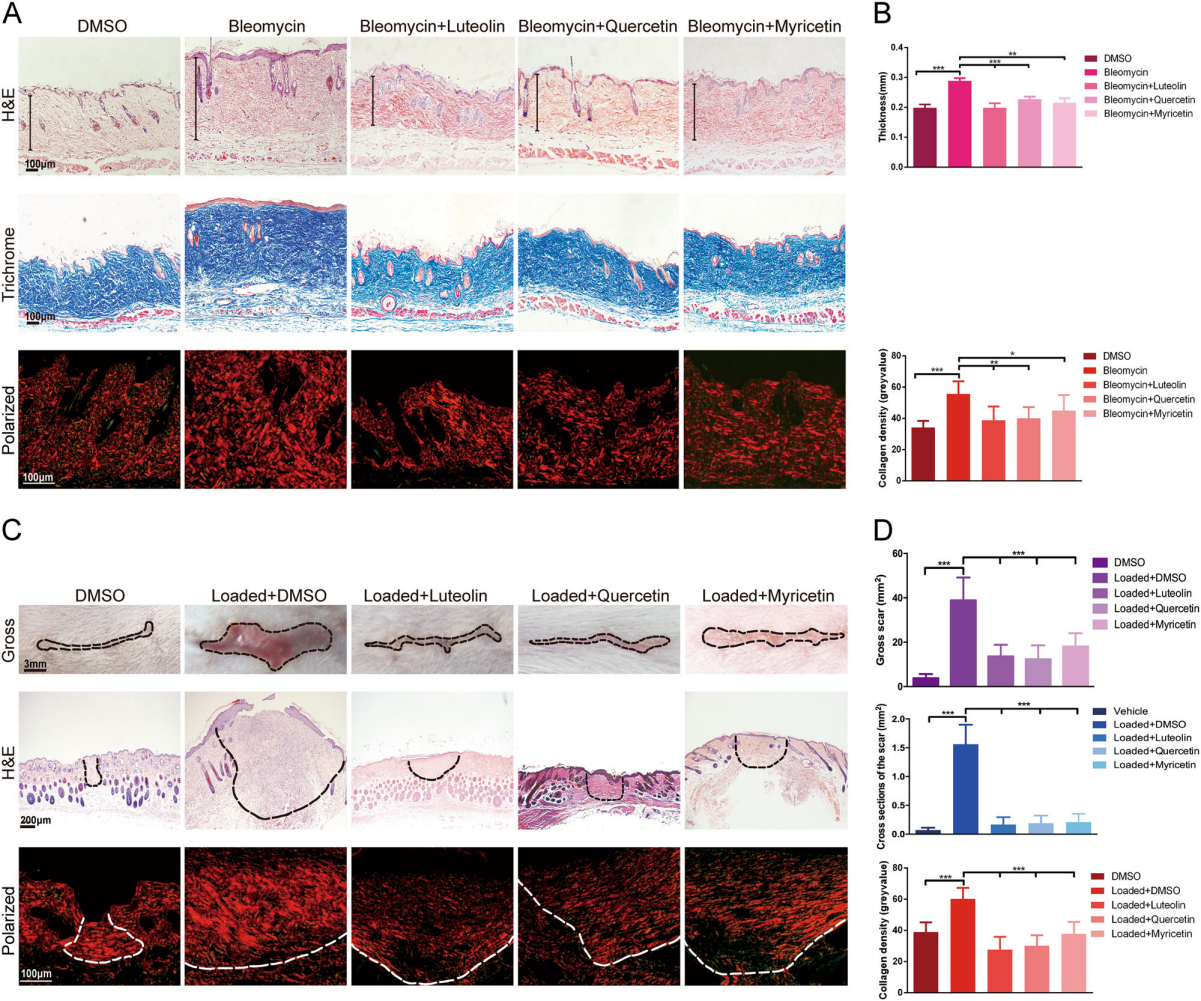
Luteolin, quercetin or myricetin ameliorates skin fibrosis and excessive collagen deposition in vivo. **a** Representative images of fibrosing skin tissue in bleomycin-induced skin fibrosis model from DMSO-treated mice and drug-treated mice after hematoxylin and eosin (H&E), Masson’s trichrome and Picrosirius red staining. **b** Quantitative analysis of dermal thickness and collagen density of fibrosing skin from vehicle-treated mice and drug-treated mice in bleomycin-induced skin fibrosis model. **c** Representative images of macroscopy, H&E and Picrosirius red staining of hypertrophic scar (HS) tissue in mechanical load-induced HS model from DMSO-treated mice and drug-treated mice. **d** Quantitative study of gross scar, cross-section area, and collagen density of HS tissue from DMSO-treated mice and drug-treated mice in HS model. Data are the mean ± SD (three independent experiments); *P *<* 0.05, **P *<* 0.01, ***P *<* 0.001

Given the results above that luteolin, quercetin or myricetin inhibited markedly the expression of collagen in vitro, bleomycin-induced skin fibrosis model and mechanical load-induced HS model, two confirmed animal models of skin fibrosis, were used to investigate the antifibrotic effect of the compounds in vivo. Histological analysis demonstrated that daily injection of luteolin, quercetin or myricetin at 10 μM reduced significantly the dermal thickness in the bleomycin model, and the macroscopic scar area and cross-section area in the loaded HS model (Fig. 2a−d). Next, we quantified collagen density using Picrosirius red staining. This revealed a lower collagen density in mice with luteolin, quercetin or myricetin treatment vs. dimethyl sulfoxide (DMSO) treatment (Fig. 2a−d). These findings identified the in vivo potency of luteolin, quercetin or myricetin to inhibit collagen expression and ameliorate skin fibrosis.

### Luteolin, quercetin or myricetin suppresses the proliferation, activation and contractile behavior of dermal fibroblasts

**Fig. 3:**
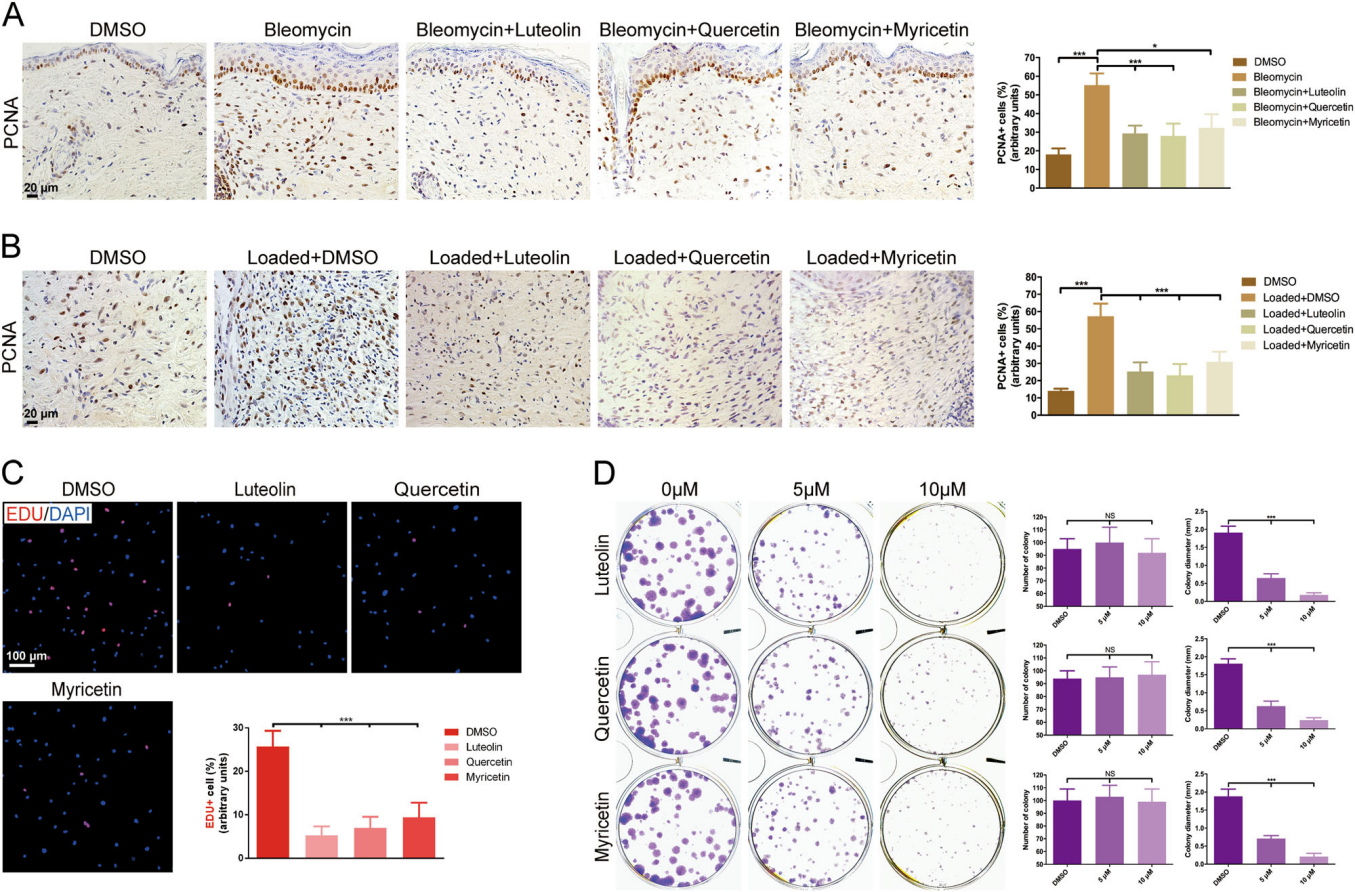
Luteolin, quercetin or myricetin inhibits fibroblast proliferation in vivo and in vitro. Representative images of hypertrophic scar (HS) in **a** fibrosing skin in bleomycin-induced model and **b** mechanical load-induced HS model from DMSO-treated mice and drug-treated mice stained for proliferating cell nuclear antigen (PCNA) and quantitative analysis of PCNA-positive cells. **c** Representative immunofluorescence images and quantitative results of EdU incorporation assay in human dermal fibroblasts (HDFs). EdU is shown by red fluorescence and nucleus by blue fluorescence. **d** Representative images and quantitative results of colony formation assay exhibit an evidently reduced number of HDF colony (purple) after drug treatment. Data are the mean ± SD (three independent experiments); *P *<* 0.05, ***P *<* 0.001

As fibroblast proliferation is closely associated with skin fibrosis formation^25^, we further investigated whether luteolin, quercetin or myricetin regulated fibroblast proliferation. Immunohistochemistry staining and quantification results showed that the agents induced a marked decrease of proliferating cell nuclear antigen (PCNA)-positive cell percentage in the dermis in both in vivo models (Fig. 3a, b). Moreover, in vitro growth of cultured HDFs was significantly retarded after luteolin, quercetin or myricetin treatment as EdU-positive cell percentage dropped significantly in EdU incorporation assay and the Gentian Violet-stained colonies reduced obviously in colony formation assay (Fig. 3c, d).

**Fig. 4:**
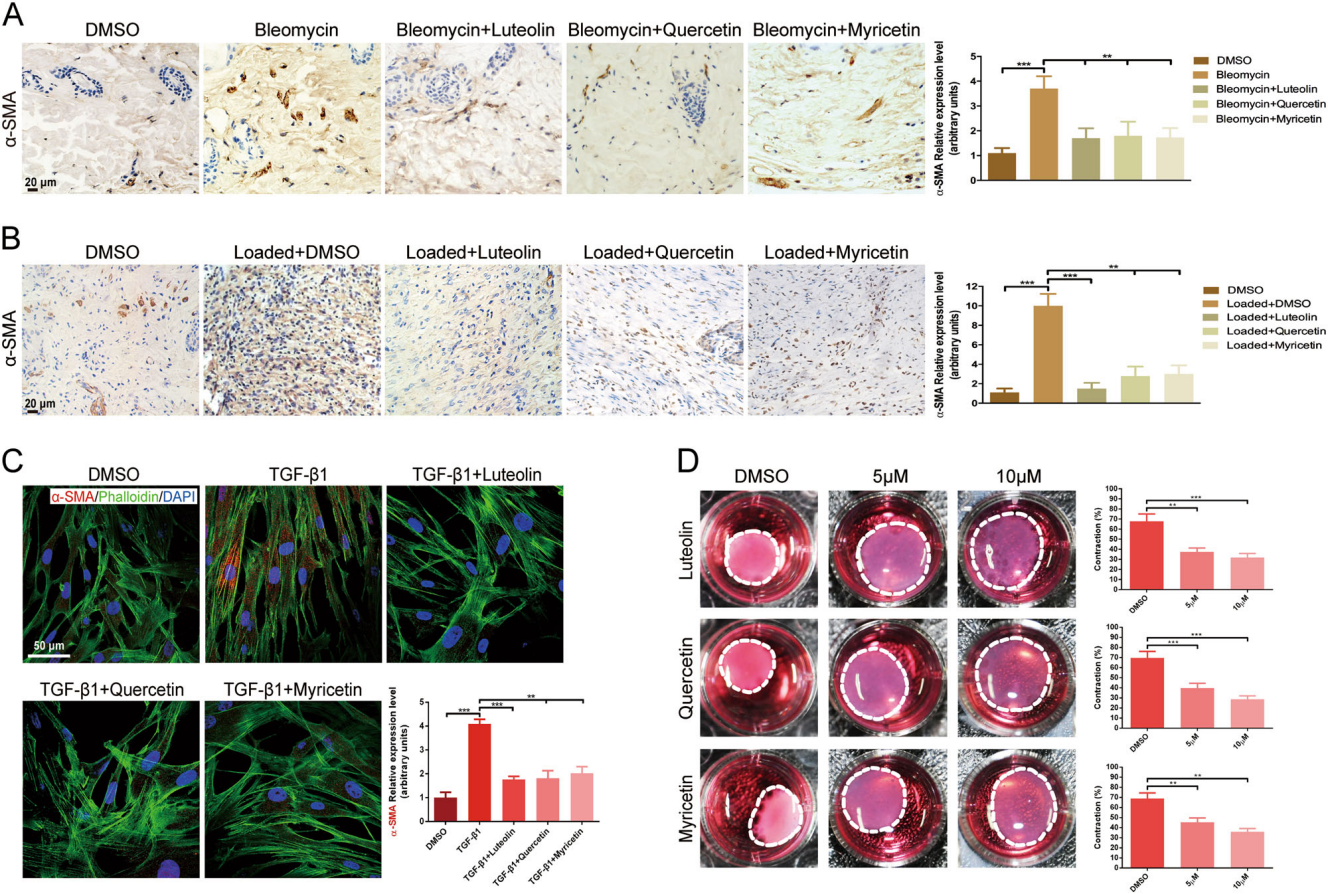
Luteolin, quercetin or myricetin suppresses the activation and contractile behavior of dermal fibroblast. Representative images and quantitative analysis of fibrosing tissues from in vivo models (**a** bleomycin-induced model; **b** mechanical load-induced model) stained for α-SMA show significant suppression induced by luteolin, quercetin or myricetin on fibroblast activation. **c** Immunofluorescence staining for α-SMA and F-actin in cultured human dermal fibroblasts (HDFs) after incubation with TGF-β1 (5 ng/ml) and DMSO or drugs for 48 or 72 h. α-SMA is shown by red fluorescence and F-actin by green fluorescence. **d** Representative images and quantitative results of collagen gel contraction assay show that luteolin, quercetin or myricetin significantly inhibited HDF-mediated gel contraction with exogenous TGF-β1 (5 ng/ml) treatment. Data are the mean ± SD (three independent experiments); **P *<* 0.01, ***P *<* 0.001

Activated fibroblasts, also called myofibroblasts, play a crucial role in ECM deposition and cicatricial contraction in fibrosing disorders^25^. Our data demonstrated that the activation of dermal fibroblasts in both animal models was notably diminished with the application of the compounds as a drug-induced decline of α-SMA expression level was shown by immunohistochemistry (Fig. 4a, b). Luteolin, quercetin or myricetin also led to significantly decreased TGF-β1-induced (5 ng/ml) α-SMA expression in cultured HDFs based on immunofluorescence staining (Fig. 4c). Furthermore, HDFs-mediated collagen gel contraction was evidently inhibited by administration of luteolin, quercetin or myricetin at both 5 and 10 μM (Fig. 4d) before TGF-β1 (5 ng/ml) application. In summary, each of the three compounds demonstrated marked inhibition on the exuberant proliferation and activity of dermal fibroblasts in vitro and in vivo.

### Luteolin, quercetin or myricetin dose-dependently inhibits the phosphorylation of Smad2/3

**Fig. 5:**
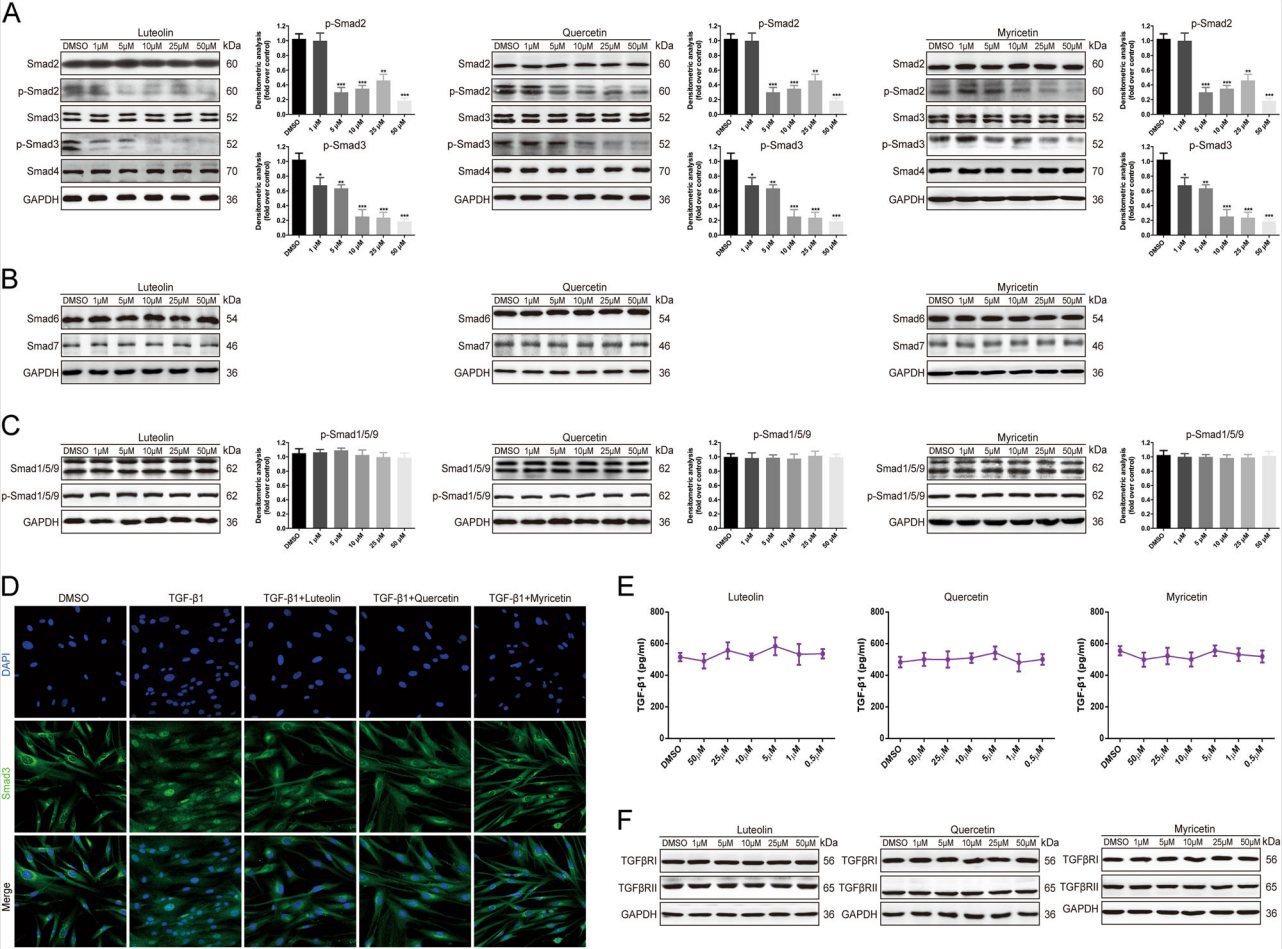
Luteolin, quercetin or myricetin downregulates the phosphorylation of Smad2/3. **a**−**c** Western blotting and densitometric analysis demonstrate that luteolin, quercetin or myricetin dose-dependently inhibits the expression of phosphorylated Smad2/3 without affecting the other factors in TGF-β/Smads signaling pathway or Smad1/5/9 in BMP signaling. **d** Representative images of immunofluorescence staining of human dermal fibroblasts exhibit a significant reduction in TGF-β1-induced Smad3 (green) translocation into the nuclei. **e** Luteolin, quercetin or myricetin has no significant impact on the expression of TGF-β1 based on ELISA assay. **f** Luteolin, quercetin or myricetin does not induce notable effect on the expression of TGFβRI or TGFβRII according to western blotting. Data are the mean ± SD (three independent experiments); *P *<* 0.05, **P *<* 0.01, ***P *<* 0.001

Since luteolin, quercetin, and myricetin exhibited significant potency in ameliorating skin fibrosis formation and fibroblast malfunction, we further looked into the underlying biomolecular mechanisms. It has been reported intensely that the prolonged activation of TGF-β/Smad2/3 pathway contributes critically to collagen deposition and fibroblast malfunctioning^14^. Interestingly, we found that luteolin, quercetin or myricetin downregulated the level of phosphorylated Smad2/3 (p-Smad2/3) in a dose-dependent manner without having significant impact on total Smad2/3 expression (Fig. 5a). Also, reduction of TGF-β-induced nuclear translocation of Smad3 in HDFs was exhibited by immunofluorescence (Fig. 5d). Moreover, we studied other Smads that modulate BMP signaling and might interact with TGF-β/Smad2/3 signaling, where the total and phosphorylated Smad1/5/9 proteins were not regulated by the compounds (Fig. 5c).

To investigate the initiation of suppressed Smad2/3 phosphorylation, the downstream and upstream factors of p-Smad2/3 were analyzed. We found that the three compounds did not affect the expression of the pathway inhibitors Smad6/7 (Fig. 5b). Considering that Smad3 forms a complex with Smad4 before translocated to the nuclei and regulating transcription, the protein level of Smad4 in HDFs was also determined by western blotting. However, it was also not obviously affected by luteolin, quercetin or myricetin (Fig. 5a). Lastly, molecules upstream of p-Smad2/3 in TGF-β/Smad2/3 signaling were studied. It was found that neither the secretion of the ligand TGF-β1 (Fig. 5e) nor the expression of the receptors TGFβRI/II was notably altered by luteolin, quercetin or myricetin (Fig. 5f).

In short, we found that luteolin, quercetin or myricetin induced a significant inhibition of Smad2/3 phosphorylation in a dose-dependent manner. However, such inhibition was not attributed to the changes in Smad1/5/9, Smad6/7, TGF-β1 or TGFβRI/II expressions. Thus, the effect of the compounds on p-Smad2/3 downregulation could be attributed to the altered function of the aforementioned molecules.

### Flavones with 5, 7, 3′, 4′ hydroxy substitution binds to the catalytic region of ALK5

During the transduction of TGF-β/signaling, Smad2/3 binds to the L45 loop of ALK5, and is then phosphorylated by the kinase^26^. Increasing evidence has shown that a variety of small molecule compounds may bind to ALK5 and impair its interaction with Smads^27,28^. Thus, we next observed the binding affinities of the selected positive compounds and a proportion of negative compounds with ALK5.

**Fig. 6:**
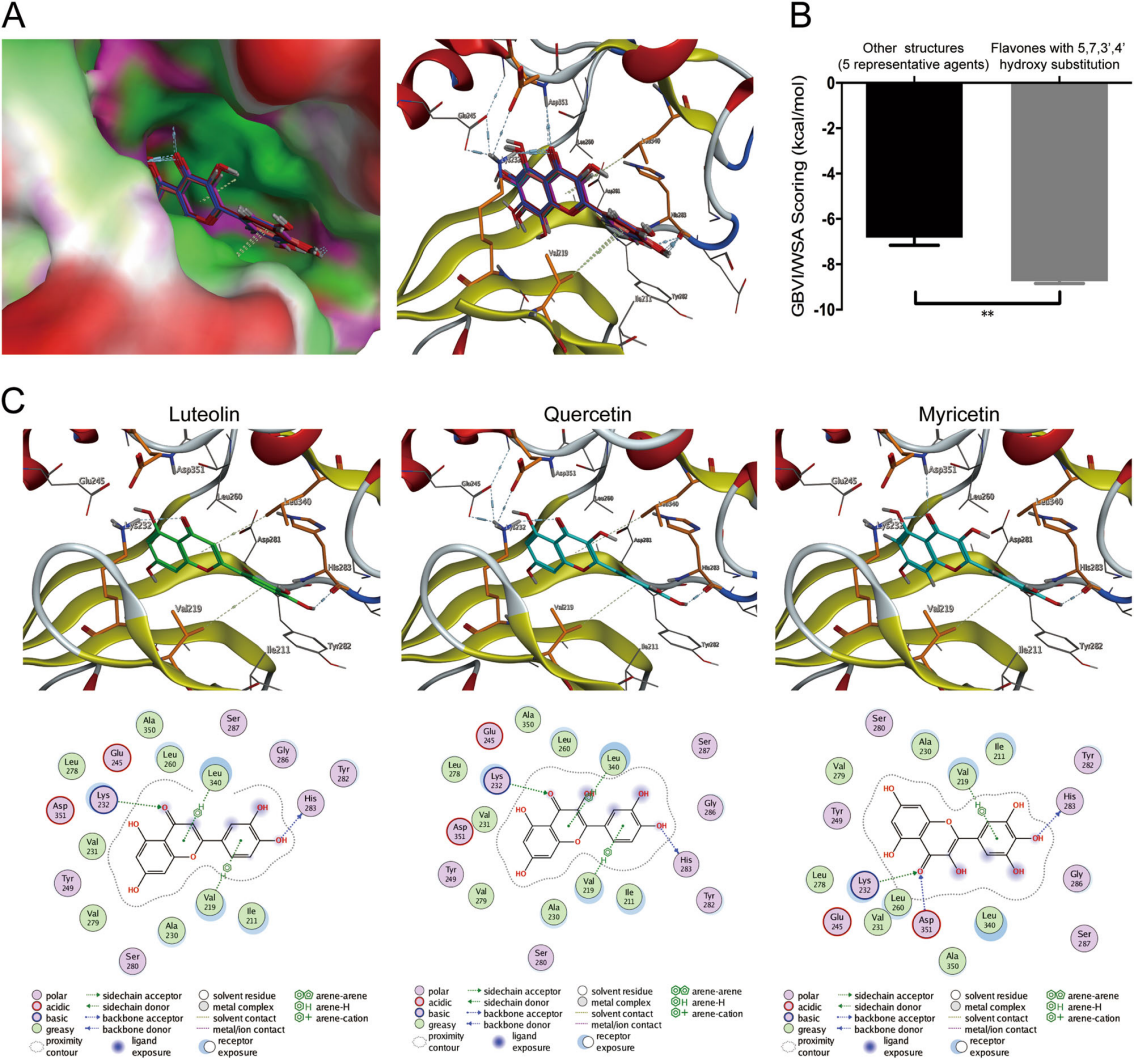
5, 7, 3′, 4′ hydroxyl-containing flavones selectively bind to activin receptor-like kinase 5 (ALK5). **a** Schematic of computational docking simulation of 5, 7, 3′, 4′-hydroxylated flavones interaction with the ATP-binding site of ALK5. Amino acid residues interacting with the compounds are shown (yellow) with the hydrogen bonds indicated by dashed lines. **b** Mean docking score of 5, 7, 3′, 4′ hydroxyl-containing flavones vs. flavones with other structures. **c** Docking simulation of luteolin, quercetin, and myricetin binding to the catalytic site of ALK5. Amino acid residues interacting with the compounds are indicated and hydrogen bonds are represented with straight dashed lines. **P *<* 0.01

The first class of compounds (eight representative compounds) has the same flavone core with 5, 7, 3′, 4′ hydroxy substitution (positive compounds). According to the docking results, this class of flavones can form several hydrogen bonds with the catalytic site of ALK5 (Fig. 6a). As seen in Fig. 6c, luteolin (5, 7, 3′, 4′-Tetrahydroxyflavone), quercetin (3, 5, 7, 3′, 4′-Pentahydroxyflavone), and myricetin (3, 5, 7, 3′, 4′, 5′-Hexahydroxyflavone) can both form two strong hydrogen bonds with the amino acid residues Lys232 and His283 of ALK5. In addition, two H−π interactions can form between the flavone core and the Val219, Leu340 of the ALK5, respectively (Fig. 6c). For the other five flavones in this class, they can almost form these interactions, such as hydrogen bonds between the compounds with the Lys232 and His283, H−π interactions between the flavone core and the Val219 and Leu340 (Supplementary Figure S5A). Due to these ligand−protein interactions, this class of flavones can bind efficiently to the ALK5 protein.

For the second class of flavones, they have the same flavone core as the first class of flavones but without 5, 7, 3′, 4′ hydroxy substitution. Five representative compounds of this class were not able to form a hydrogen bond with the His283 of the ALK5 protein (Supplementary Figure S5B). In addition, the two H−π interactions were not seen between the carbonyl group of the flavone core and the Asp351 of ALK5 (Supplementary Figure S5B). Furthermore, the docking scores of positive compounds were significantly lower than the scores of the second class of flavones (−8.74 kcal/mol vs. −6.82 kcal/mol, Fig. 6b). Therefore, the first class of the flavones with 5, 7, 3′,4′ hydroxy substitution has stronger affinity with the ALK5 protein than the second class of flavones.

### Luteolin, quercetin or myricetin inhibits ALK5 serine/threonine kinase activity

**Fig. 7:**
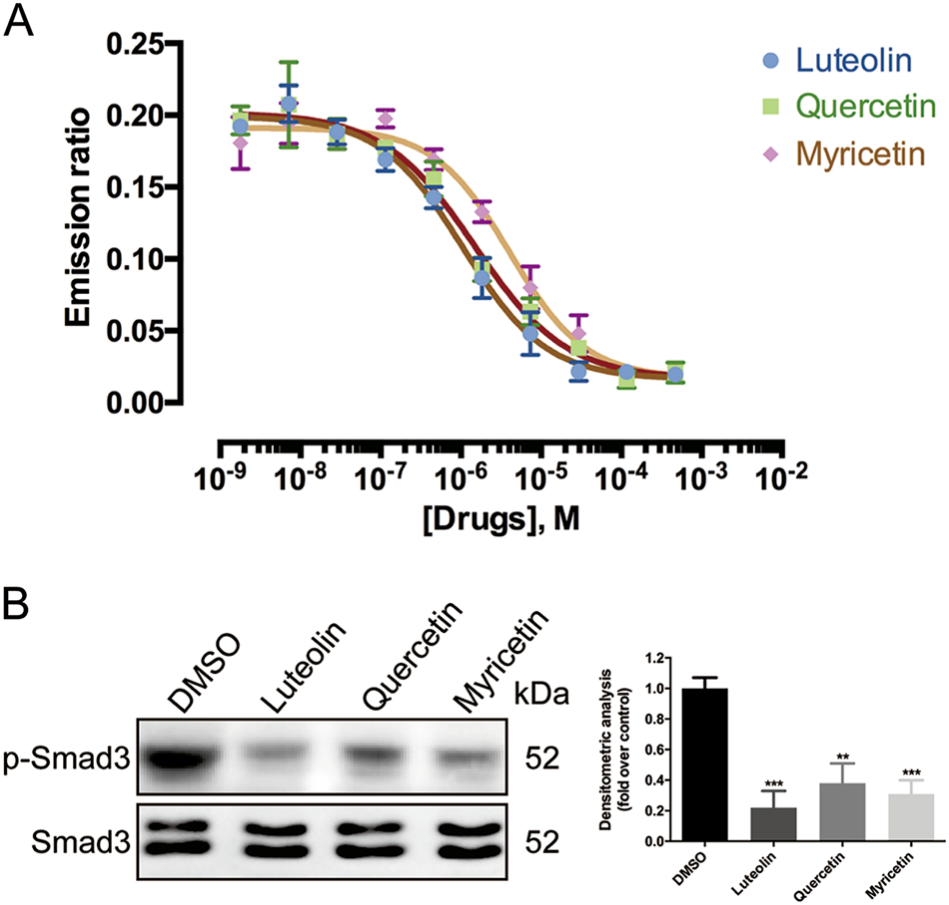
Luteolin, quercetin or myricetin binds directly to activin receptor-like kinase 5 (ALK5) and hinders ALK5-mediated phosphorylation of Smad3. **a** Dose−response curve of LanthaScreen^TM^ Eu kinase binding assay demonstrated a dose-dependent decrease in emission ratios induced by luteolin, quercetin or myricetin. Luteolin, quercetin or myricetin inhibited ALK5 activity with IC_50_ values of 1.062, 1.480, and 4.083 μM, respectively. **b** In vitro phosphorylation of GST-Smad3 by ALK5 was significantly reduced by luteolin, quercetin or myricetin. Data are the mean ± SD (three independent experiments)

In order to further validate the interaction between flavones containing 5, 7, 3′, 4′ hydroxy substitution and ALK5, we performed the LanthaScreen^TM^ Eu Kinase Binding Assay to detect competitive binding to the kinase. This assay showed a decreasing trend of the emission ratio induced by luteolin, quercetin or myricetin in a dose-dependent manner, indicating the competitive binding of the compounds with ALK5 (Fig. 7a). IC_50_ of the compounds approximated to 4 μM. Additionally, the enzymatic activity of ALK5 was significantly restrained by application of luteolin, quercetin or myricetin as ALK5-mediated production of p-Smad3 was notably decreased by the compounds in kinase activity assay (Fig. 7b).

## Discussion

Excessive collagen deposition is one of the most significant features shared by fibrotic diseases in skin^29^. In light of this, we used Col1a2 and Col3a1 as targets to perform a screen of a variety of flavonoids for candidate compounds that might be extensively effective on these diseases. It turned out that the number and position of the hydroxyl group in the compounds were major determinants of their antifibrotic property. Specifically, flavone with hydroxyls in positions 5, 7, 3′ and 4′ exhibited the most potent activity in downregulating the expression of Col1a2 and Col3a1. Thus, luteolin, quercetin, and myricetin with such structure and the most evident effect were then selected for follow-up. In vivo and in vitro studies showed efficient properties of the compounds in attenuating skin fibrosis, especially with respect to diminishing collagen production. These results indicate the potential use of the selected novel flavones in a wide range of disorders that manifest skin fibroproliferation, including HS, scleroderma and graft versus host disease.

Our investigation also discovered, at least in part, the molecular basis of flavone-induced antifibrotic effect. Analysis on fibrosis-associated signaling pathway revealed an inhibitory effect of the compounds on Smad2/3 phosphorylation, but without affecting the expression of other factors in the cascade or Smad1/5/9 in ALK1-mediated signaling. It took us to the assumption that luteolin, quercetin or myricetin might impede directly the function of the factors in signal transduction. Taking into account the increasing evidence that a variety of small molecule compounds have direct interaction with the catalytic site of kinases^30,31^, we finally performed molecular docking study, kinase binding assay and ALK5 activity assay that revealed the competitive binding of the eight representative flavones with hydroxyls in positions 5, 7, 3′ and 4′ to the catalytic site of ALK5. Hence, the present study not only confirmed the probable therapeutic effect of flavonoids in skin fibrosis, but also revealed a marked affinity between the compounds and the kinase ALK5. Thus, our findings could as well facilitate the verification of the versatile health benefits of flavonoids. For example, flavonoids are shown to have antitumor properties but few reports focus on the association between such properties and ALK5-mediated signaling^32,33^. Since ALK5 is linked with various oncogenic mechanisms including tumor angiogenesis^34,35^ and epithelial-to-mesenchymal transition^36,37^, it would be fascinating to investigate in future study whether the inhibitory effect of flavones on cancer is attributed to their binding to ALK5.

Moreover, it is of great importance that these compounds were shown to have a preference for interfering with TGF-β/Smad2/3 signaling other than other antagonistic pathways such as ALK1/Smad1/5/9 signaling, since ALK1 downregulated collagen synthesis in fibrosis-associated cells, including myofibroblasts and fibroblasts, by functioning adversely against ALK5-induced transduction^38,39^. Such specificity could enhance the antifibrotic efficacy of the compounds. In addition, one of the obstacles that hinders the clinical use of small molecule drugs is the uncertainty of side effect. It is promising that the specificity of the selected flavonoids might minimalize undesirable side effect and attain satisfactory outcomes.

The present study also emphasizes the significance of hydroxyls in flavonoids on their antifibrotic effect. It is widely acknowledged that the arrangement of the functional groups in flavonoids is critical to their biological activities such as antioxidation, hepatoprotective activities and antibacterial activities^32,40,41^. For instance, the configuration and number of hydroxyls in flavonoids is found to have profound impact on scavenging free radicals^42^. However, there have scarcely been studies concerning the association between the chemical structures of flavonoids and their antifibrotic activity. Herein, we for the first time identified the decisive functional group and configuration for the antifibrotic activity of flavonoids. 5, 7, 3′ and 4′ hydroxy substitution in flavones plays pivotal roles in inducing such bioactivity as 4′-hydroxyl forms a hydrogen bond with the amino acid residue His283 of the catalytic site of ALK5 and two H−π bonds are formed between the backbone and Val219 and Leu340. However, flavones without 5, 7, 3′, 4′ hydroxy substitution fail to form such bonds with the kinase and thus demonstrate significantly poorer affinities with ALK5. These results explain at least in part the divergent potency of flavonoids in alleviating collagen production that is predominantly regulated by ALK5/Smad2/3 signaling. Also, the findings could be constructive to the modification of the compounds in the future if necessary.

In conclusion, this study discovered a class of flavones that effectively diminishes collagen production and attenuates skin fibrosis via binding to ALK5 and thus downregulates TGF-β/Smads signaling, suggesting potential therapeutic agents for fibroproliferative disorders. More importantly, we verify that hydroxyls in positions 5, 7, 3′ and 4′ are decisive to the antifibrotic effect, which will be constructive to drug design and modification in the future.

## Materials and methods

### Cell culture

HDFs were obtained from 12 patients who had undergone scar resection 6−12 months after severe trauma or burn (Supplementary Table S1). None of the patients had received any treatment for the scar before surgical excision. All skin tissues were obtained with written informed consent according to the Declaration of Helsinki Principles. HDFs were maintained in Dulbecco’s Modified Eagle Medium (Hyclone, Thermo Fisher Scientific, Waltham, MA, USA) supplemented with 10% fetal bovine serum (Hyclone, Thermo Fisher Scientific, Waltham, MA, USA), 100 U/ml penicillin, and 100 mg/l streptomycin. HDFs were incubated at 37 °C in a humidified atmosphere with 5% CO_2_. Primary fibroblasts of passages 4–6 were used.

### Real-time PCR

The total RNA of cells was isolated using TRIzol reagent (Invitrogen, Carlsbad, CA, USA) and subjected to reverse transcription with Oligo (dT) and M-MLV Reverse Transcriptase (Thermo Fisher Scientific). Quantitative PCR amplification was performed with the ViiA™ 7 Real-Time PCR System (Applied Biosystems, Foster City, CA) by using the SYBR quantitative PCR SuperMix W/ROX (Invitrogen, Carlsbad, CA, USA). The primers used are listed in Supplementary Table S2.

### Western blotting

Cultured HDFs were lysed with radio-immunoprecipitation assay lysis buffer (Beyotime, Jiangsu, China). Protein concentrations were determined using a micro bicinchoninic acid assay (Thermo Fisher Scientific). Total proteins were separated on acrylamide gels, and immunodetected with the primary antibodies that included the following: Col1a2, Col3a1 (Genetex, Biozol, Eching, Germany, 1:1000), α-SMA, Smad6, Smad7, TGF-β receptor I (TGFβRI), TGF-β receptor II (TGFβRII) (Abcam, Cambridge, UK, 1:1000), Smad2, p-Smad2, Smad3, p-Smad3, Smad4, Smad1/5/9, p-Smad1/5/9 (Cell Signaling Technology, Beverly, MA, 1:1000). Immunoreactive bands were quantitatively analyzed with ImageJ software.

### EdU incorporation assay

Cell proliferation was detected using Click-iT® EdU Alexa Fluor® 488 Imaging Kit (Invitrogen, Carlsbad, CA, USA). In short, HDFs were incubated with EdU for 2 h, fixed in 4% paraformaldehyde (PFA), and incubated with a Click-iT EdU reaction cocktail for 30 min. Immunofluorescence signals were captured using a Zeiss 710 laser-scanning microscope (Zeiss, Thornwood, NY, USA).

### Colony formation assay

Colony formation assay of HDFs was performed using monolayer culture^43^. Cells were cultured in six-well plates at a density of 500 per well, and treated with 5 or 10 μM drugs or DMSO. After culture for 14 days, surviving colonies were fixed with 4% PFA and stained with Gentian Violet (Sigma-Aldrich, St. Louis, MO, USA) for 5 min.

### Immunofluorescence cell staining

HDFs were fixed with 4% PFA and blocked with 5% goat serum in phosphate-buffered saline with 0.1% TritonX-100 (PBST) (0.1% TritonX-100 in phosphate-buffered saline) for 1 h. For F-actin staining, cells were incubated with Alexa Fluor® 488 Phalloidin (Cytoskeleton, Inc., Denver, CO, USA, 1:200) for 1 h at room temperature. For α-SMA staining, cells were incubated with anti-α-SMA (Abcam, Cambridge, UK, 1:200) overnight at 4 °C, followed by an Alexa Fluor® 555-conjugated secondary antibody. For Smad3 staining, cells were incubated with primary antibodies against Smad3 (Cell Signaling Technology, Beverly, MA, 1:200) overnight at 4 °C, followed by an Alexa Fluor® 488-conjugated secondary antibody. Fluorescence was analyzed using a Zeiss 710 laser-scanning microscope (Zeiss, Thornwood, NY, USA).

### Collagen gel contraction assay

Collagen gel contraction assay was performed according to previous publication^44^. Briefly, 6×10^4^ HDFs and 500 μl of collagen suspension (Shengyou, Hangzhou, China) were plated per well in a 24-well plate. The plates were incubated at 37 °C for 10 min to allow collagen polymerization. After collagen gel polymerization, DMSO, luteolin, quercetin or myricetin (5 or 10 μM) were added for 1 h before TGF-β1 (5 ng/ml) treatment. Gels were detached from well and photographed at time 24 h.

### Bleomycin-induced skin fibrosis model and mechanical load-induced hypertrophic scar model

Adult female BALB/c mice aged 6 weeks were purchased from the Shanghai Slac Laboratory Animal (Slac, Shanghai, China). All procedures were approved by the Animal Care and Use Committee of Shanghai Jiao Tong University.

Skin fibrosis was induced by daily intradermal injection of bleomycin sulfate (100 μl, 1 mg/ml in vehicle) into the back skin of mice for 4 weeks as described^45^. A total of 40 mice were equally randomized into five groups: (1) Control group, where mice were injected with vehicle (100 μl sterile saline plus 1% DMSO) intradermally; (2) Bleomycin group, where mice were treated with 100 μl bleomycin sulfate; (3–5) Treatment groups, where mice were treated with 100 μl bleomycin plus luteolin, quercetin or myricetin, respectively (Supplementary Figure S6).

A mouse HS model was generated as previously published^46^. A total of 40 mice were made full-thickness incisions and divided into five groups. We established a 2-cm full-thickness incision at the dorsal midline of each mouse and then reapproximated them with 6-0 nylon sutures. Four days later, the sutures were removed, and the loading devices were fixed upon the incisions. In control group, the wound served as an internal control, without loading devices activated. In loaded group, the wound was loaded with proper function. Each of them was injected intradermally with vehicle (100 μl sterile saline plus 1% DMSO) once a day. In loaded-and-drug groups, mice with incisions that loaded and functioned well received local intradermal injection of luteolin, quercetin or myricetin, respectively. The loading devices were distracted by 4 mm every other day to maintain tension on the incisions continuously. Half of the mice in each group were sacrificed on day 14 for the sake of scar harvest while the other half on day 21 (Supplementary Figure S6).

### Histology and immunohistochemistry

Skin samples were fixed with 4% PFA, embedded in paraffin, and cut into 5-µm-thick sections. After deparaffinizing in xylene and rehydrating using an alcohol series, the sections were stained with hematoxylin and eosin (H&E), Masson’s trichrome (Trichrome stain LG solution, HT10316, Sigma-Aldrich, USA) and Picrosirius red (Fluka, Buchs, Switzerland).

For immunohistochemical staining, the sections were detected with primary antibodies against PCNA (Abcam, Cambridge, UK, 1:100) and α-SMA (Abcam, Cambridge, UK, 1:200) overnight at 4 °C. After incubation with the appropriate secondary antibodies at room temperature for 30 min, the sections were developed with diaminobenzidine and counterstained with hematoxylin. The number of positive cells was calculated with the Image Pro-Plus software.

### Molecular docking study

Three-dimensional structure of ALK5 was obtained from the Protein Data Bank (PDB code: 2WOT, 1.85 Å). The co-crystallized structure was prepared using QuickPrep module of MOE version 2016.08. The binding site for ALK5 has been well characterized based on structural information derived from the cro-crystallized structures (PDB codes: 2WOT/2WOU). The 2D structures of the two classes of flavones were built using the Builder module in MOE and converted to 3D through the energy minimization. MOE Dock module was used for docking simulation of the two classes of flavones and predicting their binding affinity with the ALK5 protein structure. The binding site of the cro-crystallized ligand was chosen as the binding pocket for docking. The GBVI/WSA dG score was used to evaluate the binding of the two classes of flavones with ALK5 protein. The binding mode and the ligand−protein interactions were analyzed in MOE after the refinement minimization.

### LanthaScreen^TM^ Eu kinase binding assay for ALK5

As a verified method to detect kinase inhibitor, LanthaScreen^TM^ Eu kinase binding assay was used to analyze the interaction of the selected flavonoids with ALK5^47^. Kinase buffer consisting of 50 mM 4-(2-hydroxyethyl)-1- piperazineethanesulfonic acid (pH 7.5), 10 mM MgCl_2_, 1 mM ethyleneglycoltetraacetic acid, and 0.01% Brij-35 was used, in which 1% DMSO was added to form compound dilution buffer. A mixture of fourfold serially diluted compounds (5 μl), kinase/antibody solution (5 μl of GST-ALK5 (15 nM) and Eu-anti-GST antibody (6 nM); Invitrogen, Carlsbad, CA, USA) and kinase tracer 178 (5 μl of 30 nM; Invitrogen, Carlsbad, CA, USA) was added to each assay well in a low-volume 384-well plate (Corning Part #3676) for 1-h incubation at room temperature. Fluorescence resonance energy transfer signal was read using a Tecan Infinite F-500 plate reader. The emission ratio was defined as the acceptor/tracer emission (665 nm) divided by the antibody/donor emission (615 nm). IC_50_ value was determined based on the sigmoidal dose−response curve that was generated using GraphPad Prism software.

### ALK5 kinase activity assay

The in vitro ALK5 kinase activity assay was performed as previously described^48^. ALK5 were obtained from Invitrogen (Carlsbad, CA, USA). Briefly, a mixture of ALK5 (2 μg) and bacterially expressed GST-Smad3 (50 mg/ml) in kinase buffer (pH 7.4; 5 mM Tris, 1 mM MgCl_2_, and 0.1 mM CaCl_2_) containing 50 mM ATP was treated with or without the selected flavonoids, and was then incubated for 45 min at room temperature. Phospho-Smad3 and GST-Smad3 in the products were detected by western blotting.

### Statistical analysis

To determine significant differences between groups in the case of count data, *χ*^2^ test was performed. In the case of continuous data, differences between the two groups were analyzed through the Student’s *t* test. Continuous data were reported as mean ± standard error (SD). The statistical analyses were performed with the statistical software package SPSS 23.0 (IBM, USA). *P* values less than 0.05 were considered statistically significant.

## Supplementary information

Supplementary Figure S1

Supplementary Figure S2

Supplementary Figure S3

Supplementary Figure S4

Supplementary Figure S5

Supplementary Figure S6

Supplementary Figure legends

Supplementary Tables
