# Supplementary figures and images for: Flavones hydroxylated at 5, 7, 3′ and 4′ ameliorate skin fibrosis via inhibiting activin receptor-like kinase 5 kinase activity
Source: Cell Death Dis. 2019 Feb 11;10(2):124. doi: 10.1038/s41419-019-1333-7 (PMC6370799; doi:10.1038/s41419-019-1333-7)

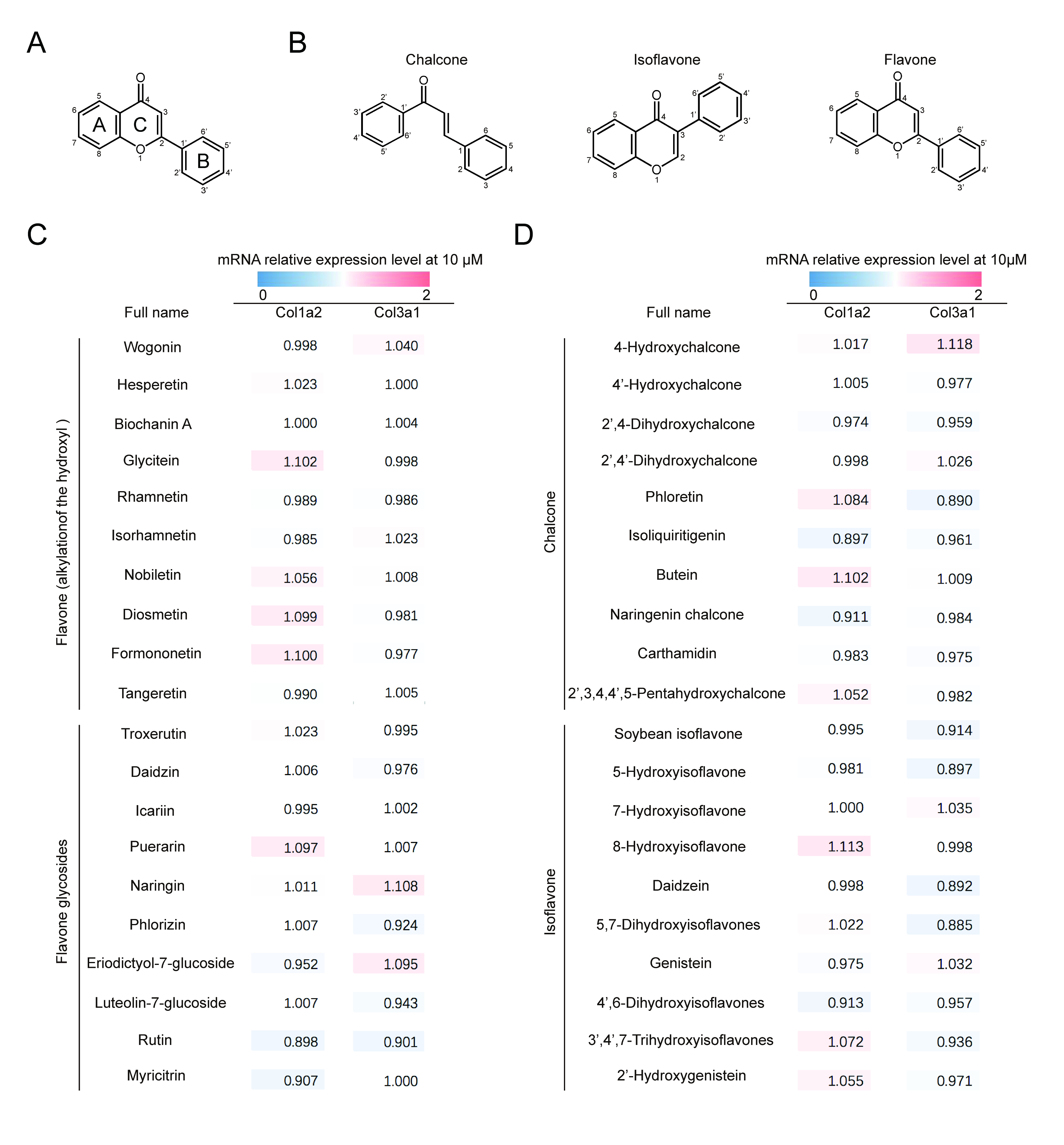

Supplement: Supplementary file 1 — Supplementary Figure S1 [file 41419_2019_1333_MOESM1_ESM.tif]

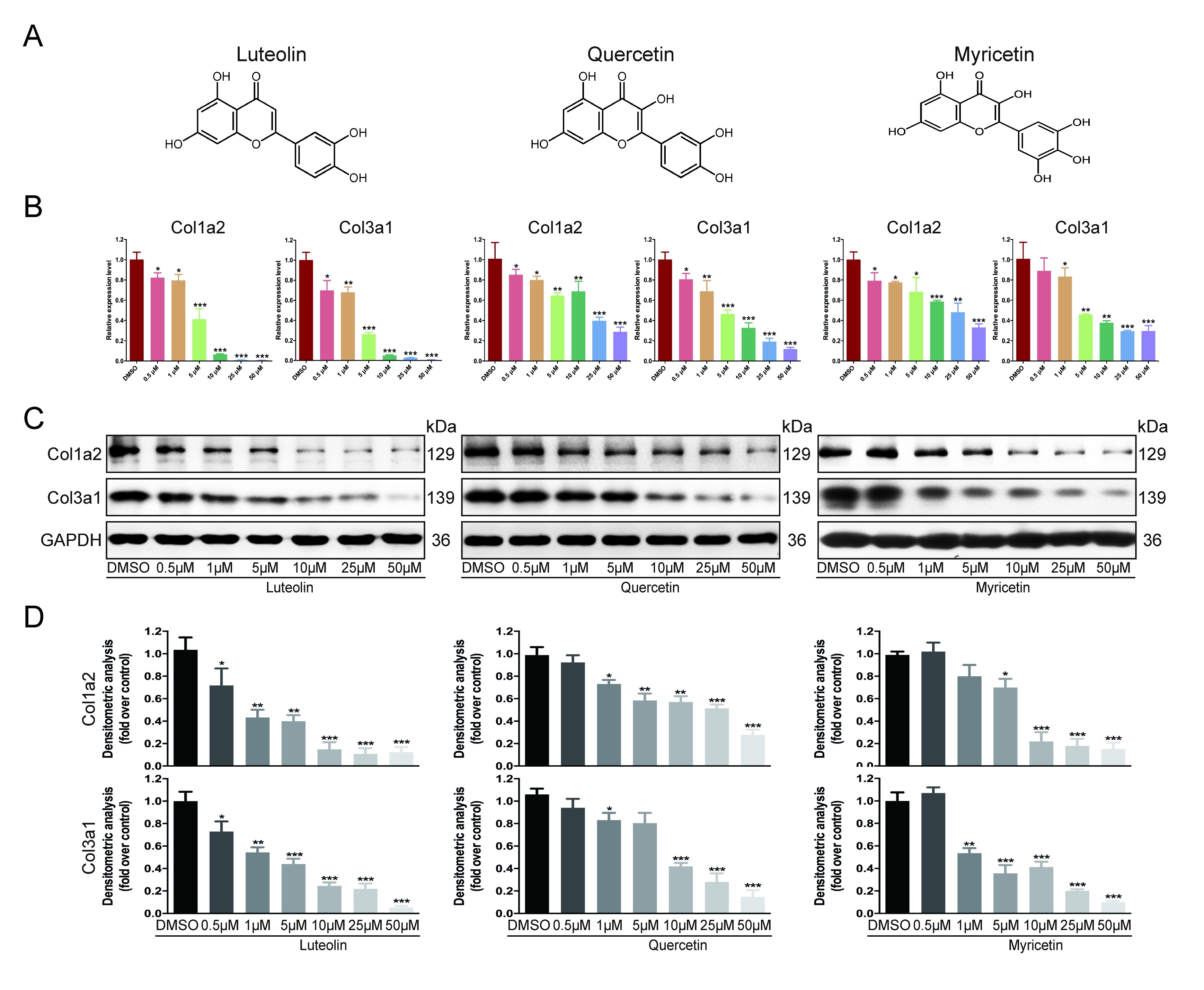

Supplement: Supplementary file 3 — Supplementary Figure S3 [file 41419_2019_1333_MOESM3_ESM.tif]

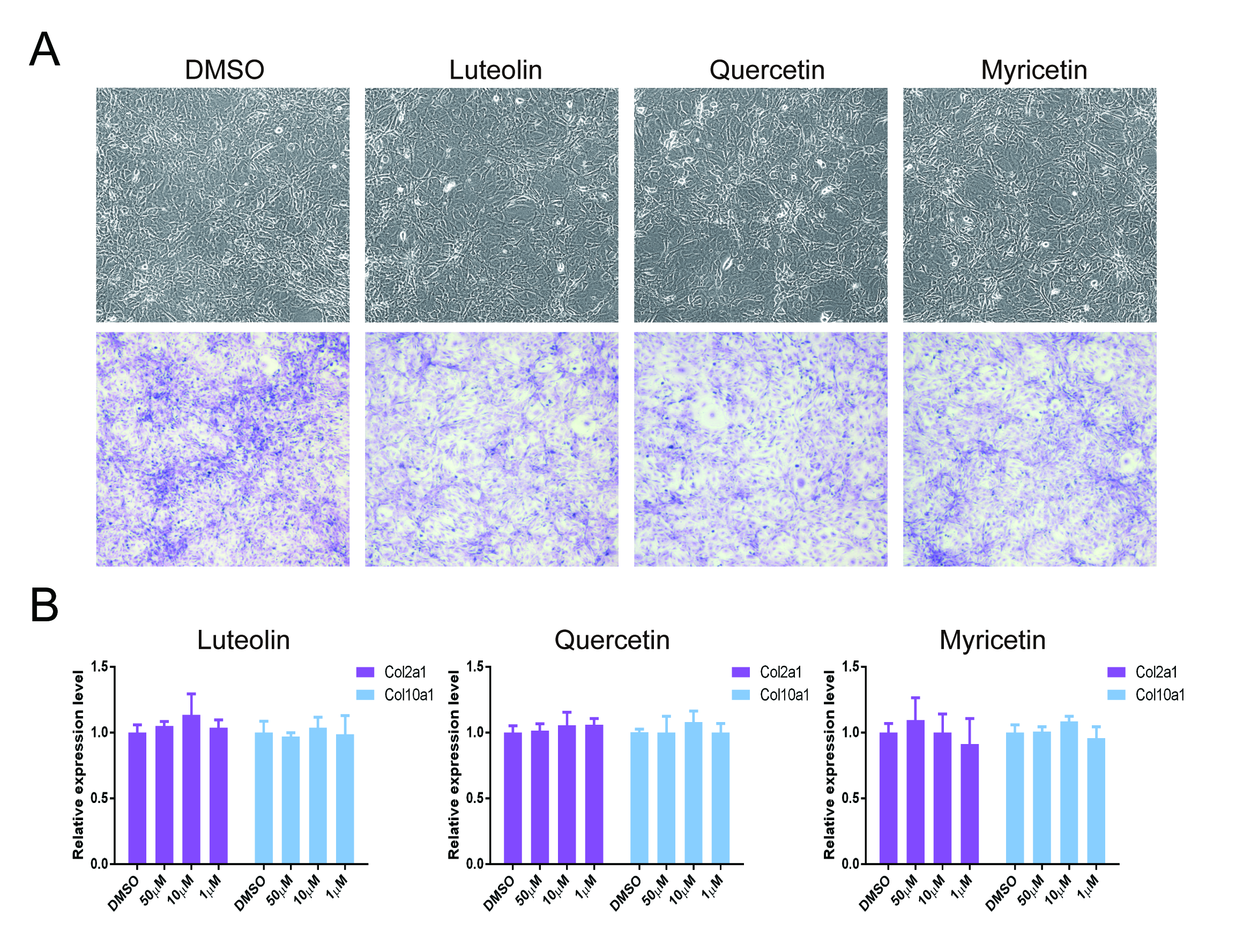

Supplement: Supplementary file 4 — Supplementary Figure S4 [file 41419_2019_1333_MOESM4_ESM.tif]

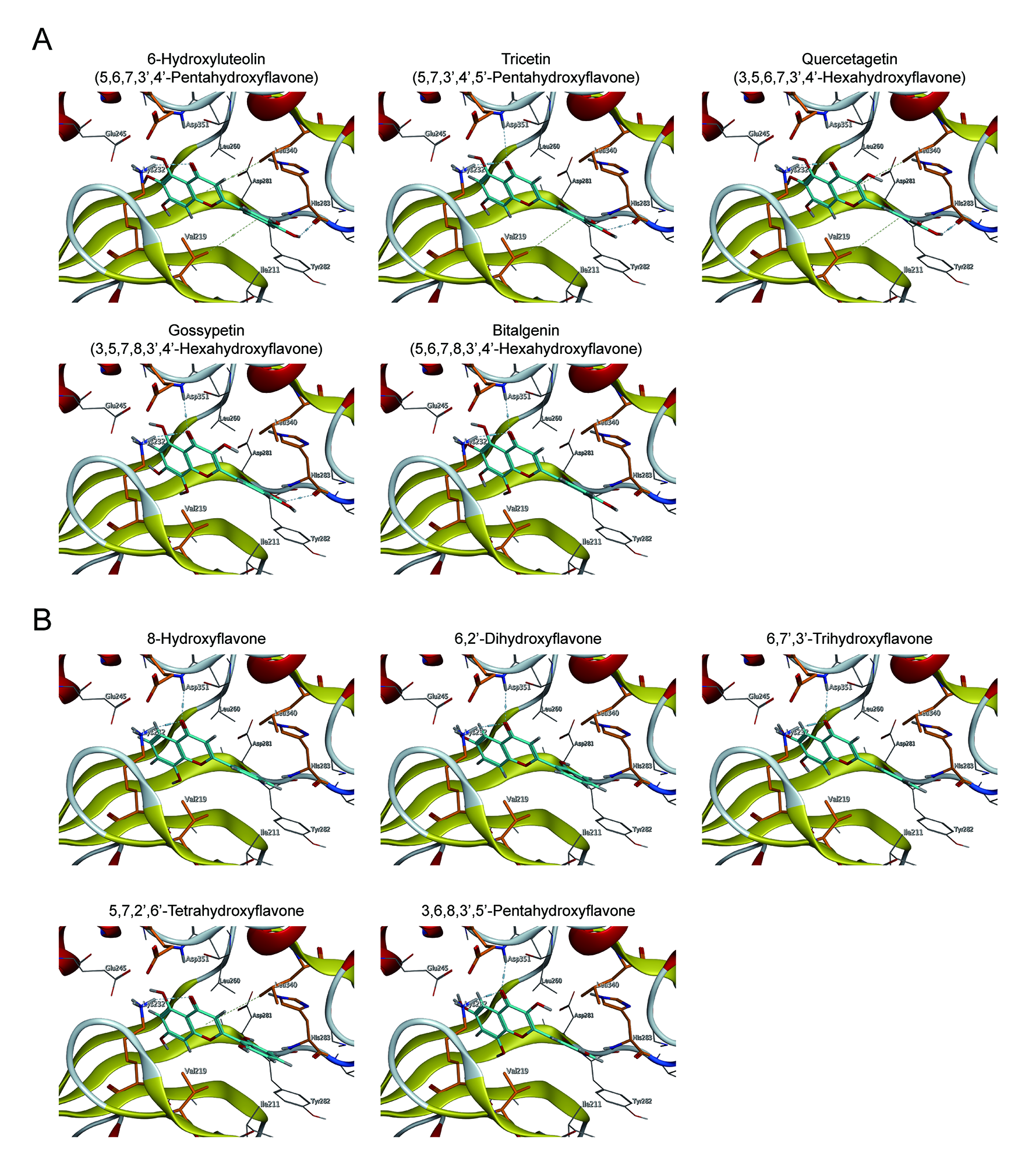

Supplement: Supplementary file 5 — Supplementary Figure S5 [file 41419_2019_1333_MOESM5_ESM.tif]

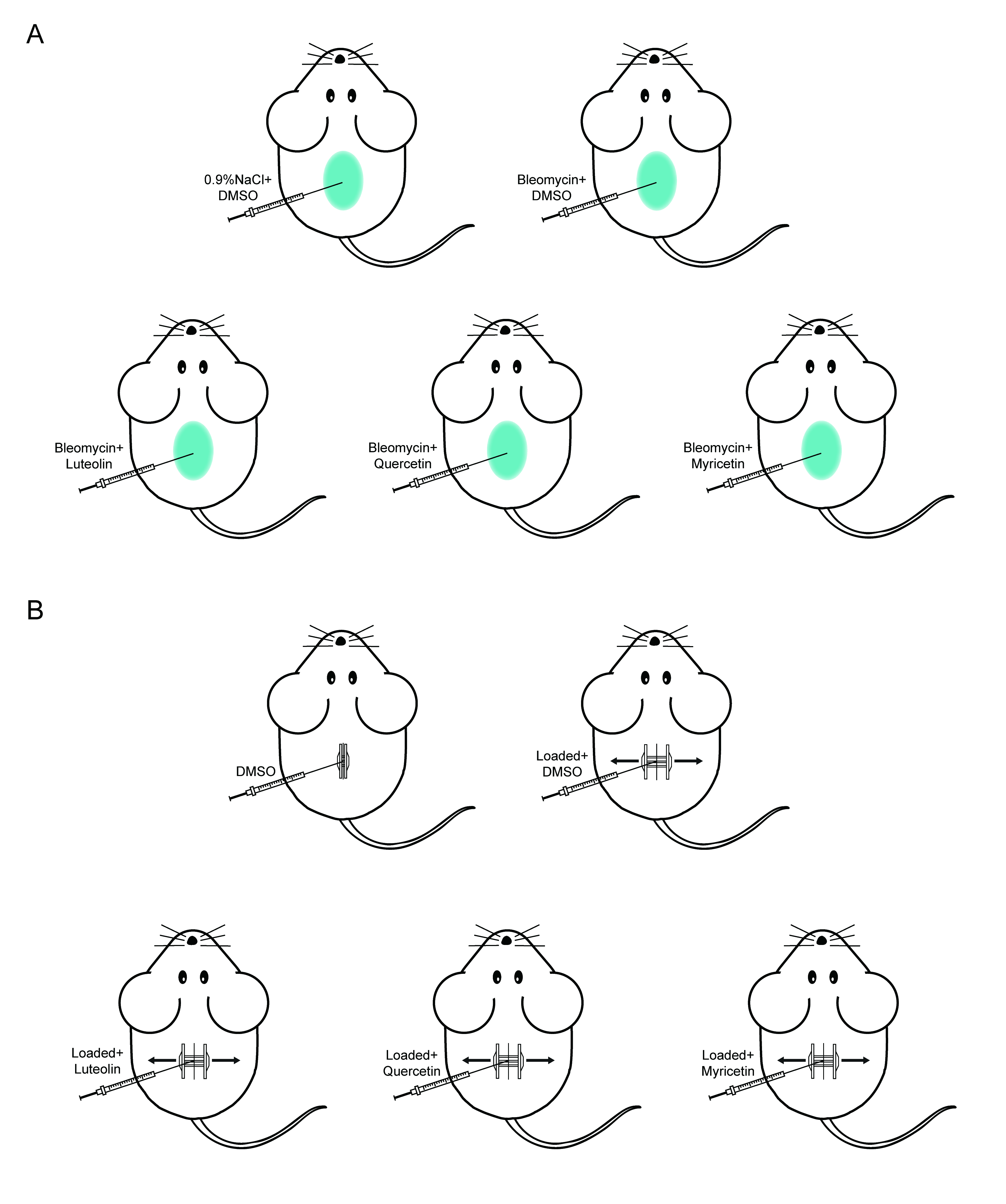

Supplement: Supplementary file 6 — Supplementary Figure S6 [file 41419_2019_1333_MOESM6_ESM.tif]
