# Supplementary material for: Flavones hydroxylated at 5, 7, 3′ and 4′ ameliorate skin fibrosis via inhibiting activin receptor-like kinase 5 kinase activity
Source: Cell Death Dis. 2019 Feb 11;10(2):124. doi: 10.1038/s41419-019-1333-7 (PMC6370799; doi:10.1038/s41419-019-1333-7)
Supplement: Supplementary file 7 — Supplementary Figure legends [file 41419_2019_1333_MOESM7_ESM.doc]

Supplementary Figure legends

Supplementary information, Figure S1. Screening of a variety of flavones, isoflavones and chalcones using Col1a2 and Col3a1 as targets. (A, B) Chemical structures of the backbone of flavone, isoflavone and chalcone. (C) Both alkylation and glucuronidation of the hydroxyl groups decrease the capacity to suppress Col1a2 and Col3a1 expressions in human dermal fibroblasts (HDFs) as is indicated by qPCR. (D) Relative mRNA levels of Col1a2 and Col3a1 of HDFs treated with chalcones and isoflavones compared with vehicle control. (three independent experiments)

Supplementary information, Figure S2. Screening of a variety of flavones using Col1a2 and Col3a1 as targets. Fold changes of mRNA expression of Col1a2 and Col3a1 in human dermal fibroblasts (HDFs) treated with hydroxyflavones (A), dihydroxyflavones (B), trihydroxyflavones (C), tetrahydroxyflavones (D), pentahydroxyflavones (E) and hexahydroxyflavones (F) versus vehicle. Data are the mean ± SD. (three independent experiments); **P* < 0.05 and the compounds were defined as positive compounds.

Supplementary information, Figure S3. Luteolin, quercetin or myricetin down-regulates the expression of Col1a2 and Col3a1 of dermal fibroblasts in a dose-dependent manner. (A) Chemical structures of luteolin, quercetin and myricetin. (B) Relative mRNA levels of Col1a1 and Col3a1 of human dermal fibroblasts (HDFs) treated with varying concentration of the compounds. (C, D) Protein levels of Col1a1 and Col3a1 of HDFs treated with varying concentration of the compounds. Data are the mean ± SD. (three independent experiments) **P* < 0.05, ***P* < 0.01, ****P* < 0.001.

Supplementary information, Figure S4. Extracellular matrix production of cultured human dermal fibroblasts (HDFs). (A) Representative images of phase-contrast microscopy and toluidine blue staining of cultured HDFs treated with TGF-β1, luteolin, quercetin, myricetin or DMSO. (B) Quantitative PCR showed no significant impact of luteolin, quercetin or myricetin on the expression of Col2a1 or Col10a1. Data are the mean ± SD. (three independent experiments)

Supplementary information, Figure S5. Docking study of other five representative flavones with 5, 7, 3’, 4’ hydroxyls and five representative flavones without such hydroxylation pattern. Schematic illustration of docking simulation of other flavones hydroxylated in 5, 7, 4’, 5’ (A) and flavonoids with other hydroxyl arrangement (B) interacting with the ATP-binding site of ALK5. Amino acid residues contacting with the compounds are highlighted and hydrogen bonds are represented with dashed lines.

Supplementary information, Figure S6. *In vivo* animal modeling and grouping. (A, B) Schematic representation of modeling, grouping and treatment protocols for two animal models (A: bleomycin-induced skin fibrosis model; B: mechanical load-induced hypertrophic scar model).
