# Supplementary material for: Flavones hydroxylated at 5, 7, 3′ and 4′ ameliorate skin fibrosis via inhibiting activin receptor-like kinase 5 kinase activity
Source: Cell Death Dis. 2019 Feb 11;10(2):124. doi: 10.1038/s41419-019-1333-7 (PMC6370799; doi:10.1038/s41419-019-1333-7)
Supplement: Supplementary file 8 — Supplementary Tables [file 41419_2019_1333_MOESM8_ESM.doc]

Supplementary information, Table S1. Patients information.

| Patients | Sex | Age (years) | Localization | Pathology |
| --- | --- | --- | --- | --- |
| 1 | Male | 12 | Shoulder | Hypertrophic scar |
| 2 | Male | 8 | Chest | Keloid |
| 3 | Female | 40 | Chest | Keloid |
| 4 | Female | 29 | Forehead | Hypertrophic scar |
| 5 | Male | 25 | Ear | Keloid |
| 6 | Female | 11 | Trunk | Keloid |
| 7 | Female | 13 | Nose | Hypertrophic scar |
| 8 | Male | 37 | Trunk | Hypertrophic scar |
| 9 | Male | 18 | Back | Hypertrophic scar |
| 10 | Male | 22 | Cheek | Hypertrophic scar |
| 11 | Female | 16 | Lower leg | Hypertrophic scar |
| 12 | Female | 27 | Back | Keloid |

Supplementary information, Table S2. Primer sequences used for qPCR analysis.

| Genes | Forward(5'to 3') | Reverse(5'to 3') |
| --- | --- | --- |
| *Col1a2* | GGCCCTCAAGGTTTCCAAGG | CACCCTGTGGTCCAACAACTC |
| *Col3a1* | TTGAAGGAGGATGTTCCCATCT | ACAGACACATATTTGGCATGGTT |
| *Col2a1* | TGGACGATCAGGCGAAACC | GCTGCGGATGCTCTCAATCT |
| *Col10a1* | ATGCTGCCACAAATACCCTTT | GGTAGTGGGCCTTTTATGCCT |
| *Acta2* | AAAAGACAGCTACGTGGGTGA | GCCATGTTCTATCGGGTACTTC |
